# Assessing the ability of bovine mesenchymal stem cells to grow on okara-based scaffolds derived from soy milk waste

**DOI:** 10.3389/fnut.2026.1828885

**Published:** 2026-08-31

**Authors:** Orit Dash, Maor Hay, Mamoun Abed El-Nabi, Shadi Tawil, Sharon Schlesinger, Kobi Meiri, Roni Rak

**Affiliations:** 1 Institute of Animal Sciences, Agricultural Research Organization - Volcani Institute, Rishon LeZion, Israel; 2 Department of Animal Sciences, Faculty of Agriculture, The Hebrew University of Jerusalem, Rehovot, Israel; 3 Tnuva – R&D Department, Rehovot, Israel; 4 The Mina and Everard Goodman Faculty of Life Sciences, Bar-Ilan University, Ramat Gan, Israel

**Keywords:** cellular proliferation, cultured meat, mesenchymal stem cell, okara (soybean residue), scaffold

## Abstract

Primary mesenchymal stem cells used in cultured meat production are anchorage-dependent, and therefore require a solid substrate for attachment and proliferation. However, widely used commercial microcarriers are typically composed of synthetic polymers, creating a significant bottleneck as they require complex and costly cell-detachment processes prior to consumption. This study investigated the repurposing of okara, a by-product of soy milk and tofu production, into microcarriers for the cultivation of adipose-derived bovine mesenchymal stem cells (AD-bMSCs). We developed and characterized multiple soy-based scaffold variants by subjecting the raw material to treatments ranging from high homogenization and plasma exposure to simple freeze-drying and wet application. Comparative analysis revealed that complex processing did not improve cell performance. Instead, the minimally processed wet okara variant emerged as the most promising candidate, streamlining production by eliminating the need for drying and pre-soaking steps. In preliminary larger-volume experiments, OP-M showed promising support for AD-bMSC growth under static conditions, with performance comparable to that of commercial microcarrier controls, from day 17 onward. Cells expanded on these soy microcarriers retained their ability to proliferate, and maintained high expression of the mesenchymal surface markers CD44 and CD29. Furthermore, cell functionality was demonstrated by successful lipid accumulation following detachment from scaffolds, at levels comparable to those observed under 2D control conditions. These findings suggest that agricultural waste can be valorized into a high-value, edible bio-scaffold that supports stem cell expansion without compromising cellular characteristics. Okara-derived microcarriers hold the potential to provide a scalable and sustainable alternative to existing microcarrier systems in cultured meat production, although further research is needed to establish and optimize this platform.

## Introduction

In traditional *in-vitro* cell cultures, cells are typically grown anchored to substrates such as treated plastic or glass, or feeder cells. This anchorage allows for normal cell cycle progression and efficient cell division (1, 2). However, in the production of cultured meat (CM), there is a need to achieve high cell quantities and to grow 3D structures of varying complexities, such as steak or minced meat (3). The utilization of biocompatible scaffolds can greatly enhance CM production by facilitating high-density 3D stem cell cultivation, enabling precise regulation of desired cell differentiation, providing cellular protection against shear stress, and contributing to shaping both the structure and texture of the product (4, 5). The choice of scaffold type depends on its intended purpose, including form, solubility, and size (6). Key considerations for cell culturing include sufficient nutrient and gas exchange through the scaffold and the ability of cells to adhere to it (5). Additional important considerations for the CM industry include the safety of the materials for consumption and the low cost of their production, to be attractive to consumers. Scaffolds can be made from natural (animal or plant-derived) or synthetic polymers (5, 7, 8). Different properties of the scaffolds (material, texture, firmness, permeability of fluids and nutrients, and pore size) influence cell adherence and proliferation, and may also direct differentiation toward specific cell types (9).

Microcarriers are a promising candidate for upscaling CM cell culture, offering a surface for adherent cells with a high surface to volume ratio (6). Current microcarriers are typically small beads of about 50–300 μm and are composed of synthetic or natural crosslinked polymers (10, 11). They are used both for cell proliferation at large scale and for cell differentiation. The first microcarriers were designed from positively charged DEAE-Sephadex (a cross-linked dextran gel) (12). Other materials used for microcarriers include polystyrene (13, 14), cellulose (15), gelatin (16, 17), Alginate (18), or polygalacturonic acid (PGA) (19). These materials are often modified to enhance cell adhesion by coating them with positively charged groups, collagen or peptides that contain adhesion motifs. Some microcarriers are composed of edible materials, whereas others are not. It is possible to use non-edible scaffolds; however, this requires cell detachment after proliferation or the use of degradable or dissolvable carriers (6). Another important consideration is the geometry of the carrier, as different surfaces influence the attachment and detachment of cells (10), and some carriers are designed to protect cells from shear forces generated in bioreactors (20). Furthermore, different cell types have distinct requirements and may grow better or worse on specific scaffolds (21). Finally, different scaffold properties can affect cell differentiation and influence lineage commitment (22).

Edible scaffolds have gained increasing interest among CM researchers, as they may provide a more cost-effective solution. Edible scaffolds eliminate the need for cell detachment, thereby reducing processing steps and minimizing cell loss during harvesting. Furthermore, their presence can contribute to the final products, in terms of texture (23) and taste (24, 25). Moreover, they can add nutritional value, such as proteins and lipids. Edible scaffolds can be made from a variety of materials, including plant or animal-based proteins, lipids, polysaccharides, or synthetic polymers (26, 27). They can be processed by various methods into small particles to serve as microcarriers (28, 29), or into larger structures such as decellularized tissue of plant or animal origin (27). Another approach to reducing scaffold cost is the use of by-products of the food industry (30–36).

Soy protein-based materials have been identified as a promising option for edible scaffolds in the development of CM due to their nutritional profile and structural properties (8, 26, 37). As a source of non-animal protein, soy is rich in protein and fiber and contains essential fats, vitamins, and minerals. Several recent studies have demonstrated the use of soy in scaffolds for CM. Soy protein was used as an additive to a polysaccharide hydrogel scaffold (38), And soy protein-based scaffolds were shown to support proliferation (39) and differentiation into myotubes (40, 41). Ben-Arye et al. used textured soy protein scaffolds to support the proliferation of bovine satellite cells and differentiation into bovine muscle tissue (42), demonstrating the potential of soy as scaffold material in the CM industry.

Okara, also known as soy pulp, is a byproduct of the soy-milk and tofu industries. Okara consists mainly of dietary fibers, and of residual insoluble proteins, lipids and carbohydrates. The disposal of okara represents a considerable environmental concern due to its high organic load, which can contribute to greenhouse gas emissions and water pollution when improperly managed. Conversely, with appropriate processing strategies, okara holds substantial potential as a functional food ingredient, and can be further utilized (43).

Mesenchymal stem cells (MSCs) are multipotent cell progenitors that possess a robust capacity for self-renewal and the ability to mature into diverse specialized tissues (*inter alia*, adipose and muscle tissues) (44). Additionally, MSCs have a good balance between multipotency and ease of isolation and culture. These biological attributes make them promising candidates for the CM industry (45, 46). Although these are not the most common cells that are studied in CM field to date, they hold a great benefit because of the ease of differentiating them into fat- a major component of meat (46). Specifically, bovine MSCs (bMSCs) provide a scalable platform for producing mainly the fat and connective tissue components of meat, and possibly also muscle (47). Adipose-derived bMSCs (AD-bMSCs) are derived from the stromal vascular fraction of bovine adipose tissue. Following cell isolation, the cells can be grown and self-renewed in-vitro on polystyrene plates (48).

In this work, we aim to identify a cost-effective microcarrier that supports and enhances the proliferation of AD-bMSCs, without impairing their ability to differentiate. We developed a method to produce novel scaffolds derived from soy-milk and tofu production waste (okara). We hypothesize that okara-based microcarriers will support efficient AD-bMSCs expansion while preserving their adipogenic differentiation capacity. To test this, we characterize the fabrication of these soy pulp-based microcarriers and evaluate the proliferation and differentiation of AD-bMSCs cultured on them, with comparison to conventional scaffolds.

## Results

In this study we investigated the performance of several novel microcarriers derived from okara and compared them to commercial plastic microcarriers (Table 1).

**Table 1:** Description and comparison of the tested scaffolds.

Scaffold name	Production treatments	Administration to cells	Size (mean ± SD)	Suitability for culturing AD-bMSCs
T4	Double ground and Heat-dried	Dry, pre-soaked	273 ± 193 μm	**
4RD	T4 subjected to high-shear homogenization and spray-drying	Dry, pre-soaked	Due to incompatibility with cell growth, particle size was not tested.	*
4EA	4RD subjected to plasma treatment type 1	Dry, pre-soaked	Due to incompatibility with cell growth, particle size was not tested.	*
4HU	4RD subjected to plasma treatment type 2	Dry, pre-soaked	Due to incompatibility with cell growth, particle size was not tested.	*
OP-FD	Freeze-dried okara	Dry, pre-soaked	266.7 ± 158.8 μm	***
OP-DC-FD	Decellularized, Freeze-dried okara	Dry, pre-soaked	186 ± 115.9 μm	*
OP-M	Okara from soy milk production, diluted 1:10 in DDW	Wet, added directly to culture	176.6 ± 116 μm	***
OP-T	Okara from tofu production, diluted 1:10 in DDW	Wet, added directly to culture	735 ± 528 μm	***
S1	Collagen-coated polystyrene microcarrier (Sartorius)	Dry, Sterilized in PBS before use	125–212 μm	***

*Low suitability **sufficient suitability *** good suitability for culturing AD-bMSCs in static 24-well coated plate, based on viability measurements. The table summarizes production treatment that was done to each scaffold, administration to cells (describes state of scaffolds after production- dry/wet and treatment before addition to culture-presoaking/sterilization/none), particle size, and suitability to support AD-bMSCs culture. Particle size was calculated from particle size analyzer Mastersizer 3000+ (Malvern Panalytical) results, 1 measurement per sample, SD represents the spread of particle sizes within each scaffold’s distribution.

### Variants of soy-milk waste based microcarriers

**Figure 1:**
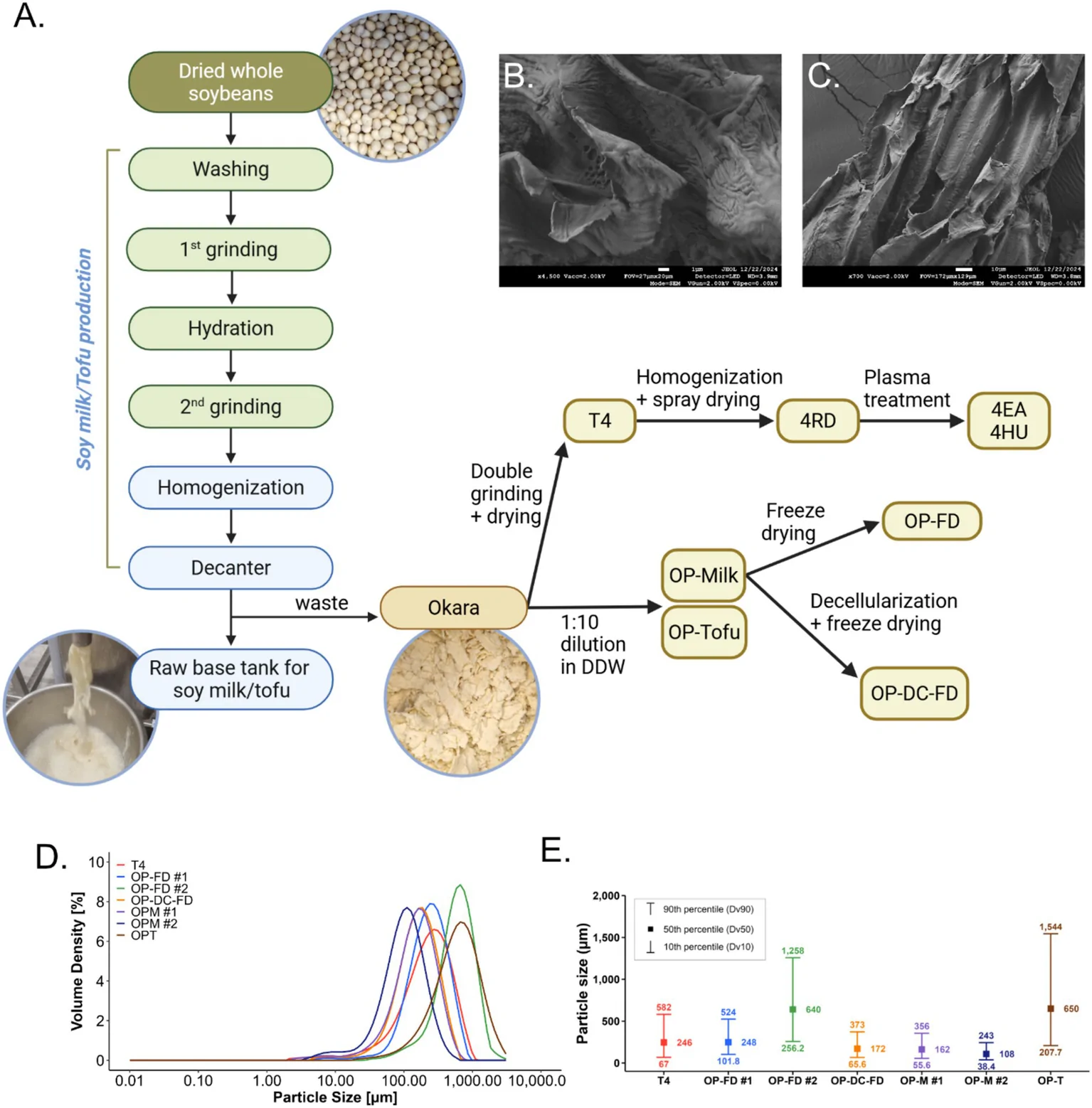
Scaffold preparation and characterization: **(A)** Soybean extraction scheme- the stages up to the decanter (including) is the process of obtaining okara, whereas rest of the steps describe the processing of the okara into different microcarriers. **(B)** A scanning electron microscope (SEM) image of T4 scaffold which underwent heat drying (bar = 1 μm). **(C)** SEM image of OP-FD scaffold which underwent freeze drying (bar = 10 μm). **(D)** Volume-based particle size distribution of dry scaffolds T4, OP-FD (2 batches), OP-DC-FD, and wet scaffolds OP-M (2 batches) and OP-T. Measurements (*n* = 1) were done using particle size analyzer Mastersizer 3,000 + (Malvern Panalytical). **(E)** Characteristic particle-size parameters of scaffolds T4, OP-FD (2 batches), OP-DC-FD, OP-M (2 batches) and OP-T, including the median particle diameter (Dv₅₀), the lowest 10% particle diameters (Dv₁₀) and highest 10% diameters (Dv₉₀) of the cumulative particle volume (*n* = 1).

The preparation of the soy-based raw material was conducted according to a staged workflow designed to generate a functional product suitable for scaffold fabrication (Figure 1A). Following homogenization, the mixture underwent primary processing, during which the liquid fraction was separated from the insoluble solids by decantation. The insoluble fraction, referred to as okara was analyzed to determine its nutrient composition (results are shown in Supplementary Table 1). The high humidity levels of an untreated okara grant it a short shelf life, due to its susceptibility to contamination. This fact could challenge industrial convenience, and therefore we tried to apply different treatments on the okara to overcome it. Distinct treatment protocols were applied to the okara in order to produce different types of okara-derived microcarriers. Eight scaffold types were evaluated alongside a commercial control. The first series comprised double-ground, heat-dried okara (T4), and three derivatives: T4 subjected to high homogenization and spray drying (4RD), 4RD with plasma treatment type 1 (4EA), and 4RD with plasma treatment type 2 (4HU). The second series included plain freeze-dried okara (OP-FD) and its decellularized counterpart (OP-DC-FD). The third series consisted of wet okara from soy-milk production diluted 1:10 in DDW (OP-M) and the equivalent from tofu production (OP-T). A commercial collagen-coated polystyrene microcarrier (S1, Sartorius) served as the reference standard.

During the production of the microcarriers, morphological measurements were performed to characterize the different scaffold types. Scanning electron microscopy (SEM) imaging was used to examine the scaffold form and structure. We show here (Figures 1B,C) the porous nature of two of the soy scaffolds tested. Quantitative particle size distribution analysis via laser diffraction demonstrated that the different processing methods generated scaffold formulations with distinct particle-size characteristics (Figures 1D,E). OP-M exhibited the smallest particle population, whereas OP-T contained the largest particles and the broadest particle-size distribution. Most scaffold formulations exhibited median particle sizes below 250 μm, whereas OP-FD batch 2 and OP-T formed a distinct coarse-particle group with substantially larger median particle sizes. Although some batch-to-batch variation was observed, particularly for OP-FD, the scaffold formulations remained clearly distinguishable based on their particle-size distributions. Overall, the scaffold formulations differed in both median particle size and distribution width, demonstrating that the different processing methods and generated scaffold particles with distinct physical architectures (Figures 1D,E; Supplementary Figures 1A–D).

### Evaluation of soy microcarrier variants for cell culture applications

**Figure 2:**
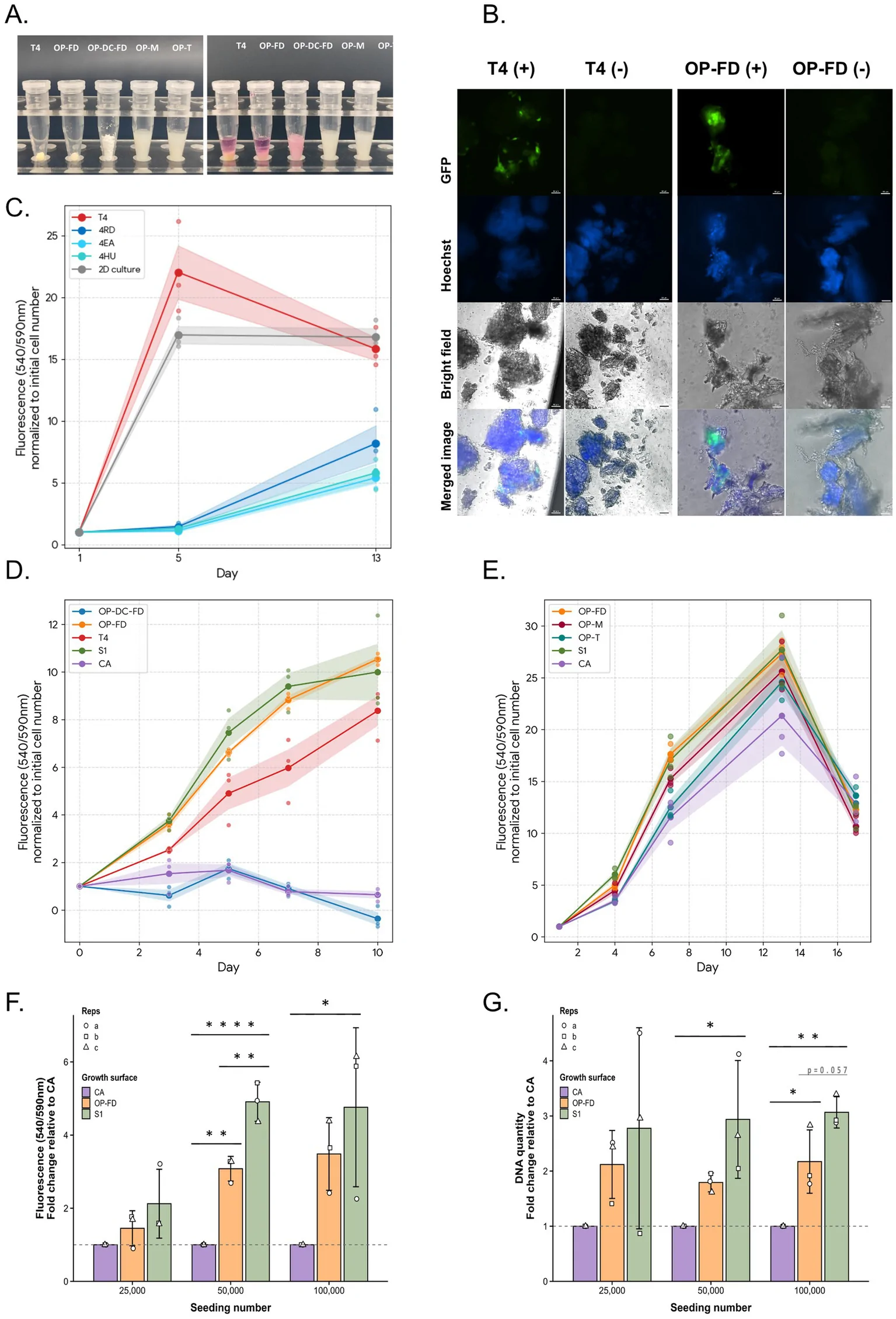
Comparison of AD-bMSCs’ viability on several types of soy microcarriers. **(A)** Photos show five types of soy scaffolds. Three scaffolds were prepared and received as dry product (T4, OP-FD, OP-DC-FD) and needed soaking of 24 h prior to cell addition, two scaffolds (OP-M, OP-T) were received in wet form. Left: Dry scaffolds, 0.01 g of each. Right: Scaffolds soaked in DMEM (in concentration of 0.05 g/mL). **(B)** Microscopy images of T4 and OP-FD scaffolds, with (+) and without (−) GFP-expressing bMSCs were obtained under similar light intensity and exposure conditions, at X20 magnitude (bar = 50 μm). Panels show GFP (green), Hoechst (blue), bright field (gray), and merged images. **(C–E)** Show three different experiments that show cell viability over time, evaluated using Alamar Blue assay. **(C)** Comparison of cell viability on variations of the T4 microcarrier: 4RD, 4EA, and 4HU toadherent cells grown in plates without splitting (2D), on two timepoints over 13 days. Sample specific blanks were subtracted from each sample, fluorescence values were normalized to initial cell seeding of 50,000 cells/well (*n* = 3, each with three technical measurements, two-way ANOVA, *p* < 0.04, complete *p*-values table is provided in supp.). **D** Comparison of cell viability over 10 days, on T4 and 2 new variants OP-FD and OP-DC-FD to viability on commercial microcarrier S1 and as cell aggregates (CA). Four timepoints were evaluated, sample specific blanks were subtracted from each sample, fluorescence values were normalized to initial cell seeding of 50,000 cells/well (n = 3, each with three technical measurements, two-way ANOVA, *p* < 0.03, complete p-values table is provided in supp.) **E** Comparison of cell viability on the microcarrier variants OP-FD, OP-M and OP-T to commercial microcarrier S1 and CA. Cell expansion was evaluated on four timepoints over 17 days. Sample specific blanks were subtracted from each sample, values of fluorescence were normalized to initial seeding density of 50,000 cells/well (*n* = 3, each with three technical measurements, two-way ANOVA, *p* < 0.04, complete *p*-values table is provided in supp.). (FG Evaluation of Alamar Blue (AB) method) by comparin g it to DNA quantification using Qubit. F Cell viability on OP-FD, S1 and as CA was measured, after 3 days of culture. Sample specific blanks were subtracted from each sample, AB measures are expressed as fold change relative to the corresponding CA condition for each seeding density (25,000, 50,000, and 100,000 cells/well). Bars represent mean ± SD, symbols indicate individual biological replicates, with three technical measurements (*n* = 3, each with three technical measurements, one-way ANOVA for each seeding density separately, *p* < 0.04, complete p-values table is provided in supp.). (*p < 0.05, **p < 0.005, ****p < 0.00005. G DNA-base) d quan tification of cell number was performed, after 3 days of culture. Sample specific blanks were subtracted from each sample, estimated cell number is expressed as fold change relative to the corresponding CA condition for each seeding density (25,000, 50,000, and 100,000 cells/well). Bars represent mean ± SD, symbols indicate individual biological replicates, with two technical measurements (*n* = 3, each with three technical measurements, one-way ANOVA, *p* < 0.02, complete p-values table is provided in supp.) *p < 0.05, **p < 0.005.

In this study, we aim to evaluate whether soy microcarriers are suitable for cell culture support. Throughout the study, we focus on AD-bMSCs (49), given their particular relevance to the CM industry (46) and their capacity to generate fat tissue, a key component which critically influences the flavor profile and textural properties of meat. To examine structural behavior, five soy scaffold variants were compared in both dry and hydrated states (Figure 2A). Despite having identical weights, the scaffolds displayed clear differences in volume. Prior to hydration, these differences stemmed from variant-specific particle forms and physical characteristics, whereas post-hydration volumes were primarily governed by their respective medium absorption capacities.

To ensure that soy microcarrier does not release materials that hamper cell growth, we performed a trans-well experiment, in which the microcarriers were separated from the cells by polyester membrane. The T4 microcarriers did not affect the AD-bMSCs without direct contact (Supplementary Figure 2A). Next, we wanted to evaluate whether the cells adhere to the scaffolds. For this purpose, we seeded GFP-expressing bMSCs on T4 scaffolds. Two days after seeding samples were moved into new wells for examination. Microscopy imaging showed co-localization of cells and scaffolds (Figure 2B) and supported the potential of cell attachment to the soy scaffolds.

Having confirmed scaffold cytocompatibility, we next assessed the capacity of each soy microcarrier variant to support cell proliferation. The first series of soy microcarriers tested, included T4 and three new scaffold types- 4RD, 4EA and 4HU (three different variants of modifications of T4, see Figure 1A and method section for details). Cells were seeded on the microcarriers in 24-well plates and grown for 13 days. Cell viability on the soy microcarriers was compared to viability of cells in 2D culture, using Alamar Blue mitochondrial assay, on days 5 and 13 (Figure 2C). Total culture viability on T4 microcarriers was significantly higher than on the other three scaffolds types, at both time points (*p* < 0.006). Viability of samples with T4 microcarriers was higher in comparison to 2D culture, on day 5 but not on day 13 (*p* < 0.03 and *p* = 0.99 respectively, full *p*-values table is provided in Supplementary data). We found that the additional treatment steps in the production protocol, did not increase the scaffolds’ ability to support cell viability, and that T4 without additional treatments, supported the cells better than the new variants. Thus, the T4 microcarriers remained a comparison standard, for further improvement of the scaffolds’ properties, ease of production, and for the improvement of cell growth protocols.

Next, a series of freeze-dried microcarriers was assessed. Cell viability on OP-FD and OP-DC-FD, was compared with cell viability on T4, with cell viability on the commercial collagen-coated microcarriers SoloHill (termed here S1, Sartorius). This scaffold was selected for comparison based on initial calibration of six commercial scaffolds (Supplementary Figures 2B,C). Also, we compared to the viability of cells that grew as cell aggregates (CA, also referred to as scaffold-free or suspension aggregate cultures), that serve as a control group since they represent the baseline behavior of anchorage-dependent cells forced to grow without an external attachment substrate. Cells were grown in 24-well plates on the different microcarriers, and their viability was evaluated, using Alamar Blue assay, 4 times during 10 days (on days 3,5,7 and 10). On measurement day, growth medium with scaffolds from each well were collected and transferred into new wells and cell viability was measured in both new and all wells. We found almost no sign for cell viability in original wells, assuring that almost all cells in the wells were not adhered to the plastic, but to scaffold or other cells. Cell viability was higher on T4 microcarriers, in comparison to OP-DC-FD and to CA, from day 5 and on (*p* < 0.002). Cell viability on OP-FD and on S1 was significantly higher than on OP-DC-FD on all time points, and higher than viability as CA, from day 5 and on (*p* < 0.004). The viability on OP-FD and on S1 was mostly similar (*p* > 0.99), except for day 7, on which the viability of the cells on OP-FD was significantly lower than the viability on S1 microcarrier (*p* = 0.009). OP-FD supported higher viability rate in comparison to T4, although it was significant only on day 7 (*p* = 0.009), the difference is very apparent on rest of time points. Considering the somewhat easier production protocol that OP-FD requires, we chose OP-FD to be our new standard for comparison (Figure 2D). In addition, microscopy imaging showed colocalization of cells and OP-FD scaffolds, supporting that cell adhere to the scaffolds (Figure 2B).

Finally, we evaluated cell viability on plain diluted sterilized okara. OP-M originated from soy-milk industry, and is the precursor of OP-FD and OP-DC-FD. While, OP-T originated from tofu industry. We compared cell viability on OP-M, OP-T, OP-FD, S1 and as CA (Figure 2E). Cells were seeded in 24-well plates with the 4 scaffolds and grown for 17 days. Alamar Blue assay was performed 4 times during this period (on days 4,7,13 and 17). There were almost no significant differences in viability rates among the conditions. The cell viability on OP-FD, OP-M, OP-T and S1 was similar (*p* > 0.15). The OP-T microcarriers were not convenient to work with, due to relatively large particle size (784 μm ± 43.3) that blocked pipettes. OP-FD and OP-M scaffolds support cells in a similar success, with a non-significant advantage for OP-FD; however, the OP-M microcarriers making process lacks the last stage of drying, in the OP-FD microcarriers production. This fact turned OP-M into our favorite candidate for the next experiments, as it enabled immediate addition to the cells, without prior soaking of the scaffolds for 24 h. In addition, reducing steps in the manufacturing process, results in lower scaffolds’ cost.

We found that after 17 days of culturing, cell viability in all conditions decreased (Figure 2E). This may have been caused by limited growth area within the 24-well plate (Figure 2E). To address this constraint and assess the scalability of the process, we established a higher-volume testing platform. This involved culturing the cells on soy microcarriers within larger vessels.

To ensure that Alamar blue assessment of viable cells is a valid method for comparing cell growth between the different conditions we conducted an experiment that compared results of same samples measured using Alamar blue and with Qubit which quantifies DNA content of the samples (Figures 2F,G). In both methods, there was a significant difference in cell growth on scaffolds in comparison to growth as CA in the higher seeding densities (50,000 and 100,000 cells/well). Cells grew significantly better on S1 in comparison to CA in both methods, in both seeding densities (*p* < 0.04). Cells grew significantly better on OP-FD in comparison to CA, in Alamar Blue measurement in the 50,000-seeding density (*p* = 0.001), and in Qubit measurement in the 100,000-seeding density (*p* = 0.02), conditions Alamar Blue:100,000[cell/well] and Qubit:50,000[cell/well] were not significant, but there is a clear trend that shows higher cell growth on OP-FD in comparison to CA. A strong linear relationship was observed between the two assays for all culture conditions (Supplementary Figures 3A–C). Furthermore, Alamar Blue measurements showed lower standard deviations, indicating lower variability between replicates.

### Culturing large volumes of AD-bMSCs using soy-milk waste based microcarriers

**Figure 3:**
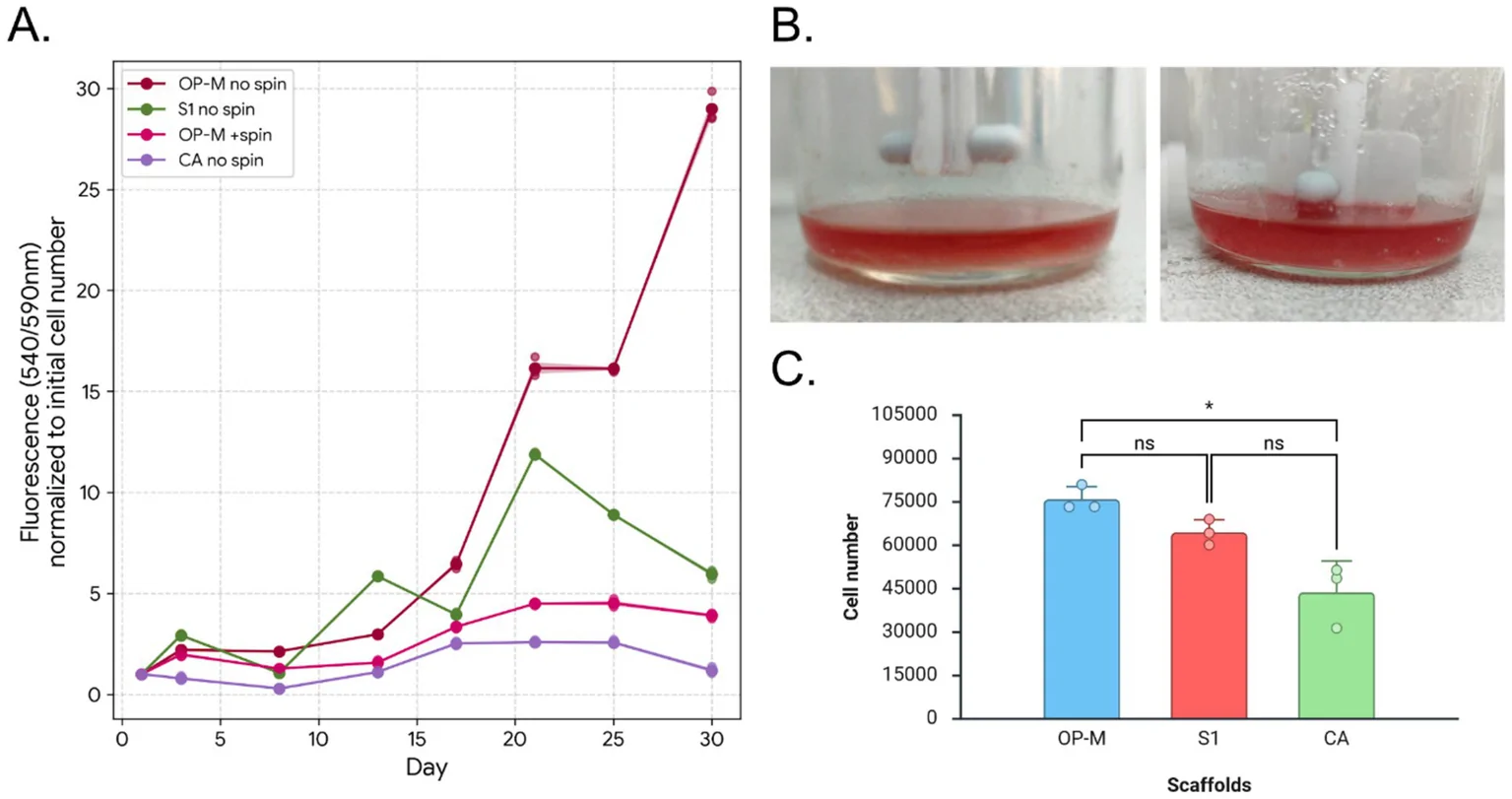
Comparison of AD-bMSCs’ viability on several types of soy microcarriers, over time in spinner flasks, and viability of these cells on plates, after descending from microcarriers. **(A)** Cell expansion over time on OP-M scaffold in comparison to a commercial scaffold (S1) and to cells that grow in aggregates (CA, without any scaffolds) was evaluated. Cells were grown for 30 days, and viability was evaluated seven times, using Alamar Blue assay. Sample specific blanks were subtracted from each sample, values of fluorescence were normalized to initial cell seeding of 50,000 cells/mL (*n* = 1, with three technical repeats). **(B)** Spinner flasks’ stirring pole position as in with (+spin) or without stirring (no spin) conditions. Left: Stirring pole placed as in the no spinning OP-M, S1 and CA conditions. Right: Spinner flasks’ stirring pole placed as in the spinning OP-M condition. **(C)** Cell growth on plates after culturing in flasks on scaffolds OP-M and S1 or as CA for 30 days. 50,000 cells/well were seeded in 6-well plates, in triplicates for each condition. Cells were counted directly after 4 days (*n* = 3, Kruskal-Wallis test, *p* = 0.022) bars represent mean ± *SD*, *p < 0.05.

Next, we wanted to test the ability of cells to grow on soy microcarriers in larger volume, a state that simulates better (than a cell culture plate) the feasibility to use the scaffolds in bioreactors, as the industry intends to do. Cells were seeded on OP-M and S1 microcarriers in 20 mL of growth medium inside 1 L glass flasks at a density of 50,000 cells/mL. Cells were grown for 30 days, and viability was evaluated seven times (on days 3, 8, 13, 17, 21, 25 and 30) using Alamar Blue assay and a cell standard curve. Cell expansion over time is shown in Figure 3A.

The cell viability on OP-M was tested both in a stationary flask (OP-M no spin), and in spinner flasks with a rotation speed of 80 rpm (OP-M with spin) and compared to stationary flasks with S1 and CA. The spinner flask configurations are illustrated in Figure 3B.

At every evaluation point, a sample of 0.5 mL was taken from each flask and cell viability was examined. In addition, 5 mL of old growth medium was removed from each flask and 7 mL of fresh medium was added, along with new microcarriers to substitute the amount that was taken for cell viability evaluation, and to keep the total concentration of scaffolds constant (0.005 g/mL). Up to day 17 there is a slow increase in cell number in all conditions, during which all samples but OP-M no spin experienced a small decline in expansion. On day 21 the number of cells on OP-M no spin and on S1 increase dramatically, while OP-M with spin and CA keep expanding slowly. After day 25 OP-M no spin maintains the rapid expansion, while all other samples collapse. In this preliminary experiment, cells on the soy microcarrier OP-M no spin showed higher Alamar Blue signal than that of cells on the commercial scaffold S1 (Figure 3A). Additional experiment or culturing cells in large volumes (different vessel type) was conducted showing similar trends, the results are presented in Supplementary Figures 4A,B.

### Cell characteristics following growth on scaffolds

Following cell expansion on the microcarriers, we sought to characterize the cells to ensure the maintenance of their MSC phenotype and functional characteristics. Cells from three conditions: OP-M no spin, CA and S1, were transferred back into plastic plates to ensure their ability to adhere to plastic. We inspected the plates under a microscope and recognized the usual cell morphology of adherent cells (Supplementary Figure 4C). We then compared proliferation rates of cells that grew on the soy microcarriers to cells that grew on commercial scaffolds and to cells that grew as CA (Figure 3C). We found that the proliferation rate of cells that were harvested from the soy microcarrier OP-M was significantly higher than of cells that grew as CA (*p* = 0.022, Kruskal-Wallis test), and did not differ significantly from proliferation rate of cells that were harvested from the commercial scaffold S1.

**Figure 4:**
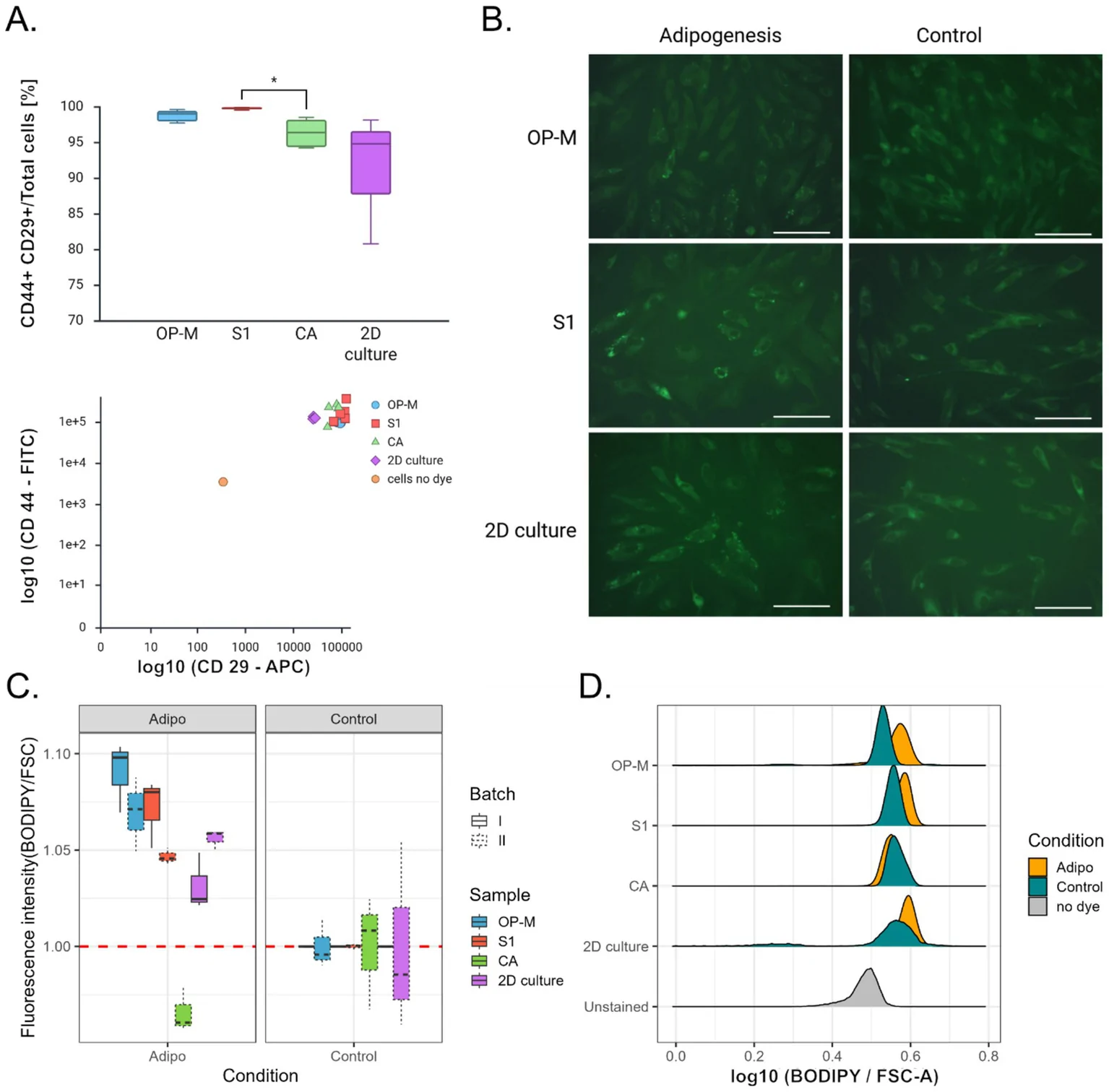
bMSCs characteristics and adipogenesis after growth on scaffolds. **(A)** Flow cytometric analysis results of the appearance CD29 and CD44 (membranal markers) on the cells. Upper plot shows the percentage of cells that show both markers relatively to total cell amount in each sample. Marker appearance in all conditions was similar to 2D culture control, and significantly different only between S1 and CA conditions (2D culture *n* = 3 other conditions *n* = 6, one-way ANOVA, *p* = 0.03), * *p* < 0.05, bars represent mean ± SD. Lower plot shows log10 expression of CD44 conjugated to FITC over log10 expression of CD29 conjugated to APC (*n* = 3, each dot represents one biological repeat). **(B)** Microscopy images of cells from 3 conditions (after growth on OP-M and S1 scaffolds and 2D plate control) after receiving adipogenesis or regular (control) medium for 2 weeks, bar = 100 μm. **(C)** Box plots show BODIPY fluorescence intensity normalized by size (FCS) of cells that received adipogenesis medium, and their controls, for 2 batches of experiment, in each: Three technical measurements, except for control of batch I with one technical measurement, CA was measured only in batch II (*n* = 2, two-way ANOVA, *p* < 0.04). **(D)** Histograms of FITC intensity normalized by cell size, for each condition of batch II.

To confirm the maintenance of the mesenchymal phenotype following scaffold-based expansion, we assessed the expression of the surface markers CD44 and CD29. The cells were incubated with FITC-conjugated anti-CD44 and APC-conjugated anti-CD29 antibodies, followed by flow cytometric analysis (Figure 4A). CD marker expression was high, and their appearance in all conditions was similar to the appearance in the 2D culture control (*n* = 3, one-way ANOVA, *p* > 0.5). S1 showed significantly higher marker expression than CA (*p* = 0.031), whereas, there was no other difference in marker expression level found.

### Cell ability to accumulate lipids, after proliferation on scaffolds

To confirm that the cells retained their adipogenic potential after expansion on the soy scaffolds, we assessed their ability to accumulate lipids. As a first test we checked the ability of the cells to accumulate oleic acid (OA). Cells that grew in flasks, with or without scaffolds maintained the ability to accumulate fat, but the difference in Bodipy FITC intensity was not significant, in comparison to control cells. Notably, the cells that were harvested from OP-M showed marginally significant higher fat accumulation in comparison to control cells (Supplementary Figures 5A,B; *p* = 0.0589). This result led us to test the ability of the cells to differentiate into adipocytes.

To evaluate lipid accumulation potential following cultivation on microcarriers, cells were subjected to adipose-differentiation protocol following 1 week of culturing on indicated microcarriers and lipid accumulation was measured. In order to be able to better quantify the lipid accumulation, we performed the protocol on cells that were removed from scaffolds and seeded attached to cell culture plates, as the scaffolds interfere with cell visualization. The cells were harvested plated in 2D plates, and subjected to adipogenesis induction medium or to growth medium as control. After 2 weeks of adipogenic induction, lipid accumulation was evaluated by fluorescent staining of fat content (BODIPY) and was visualized with microscopy (Figure 4B) and measured by flow cytometric analysis (Figures 4C,D). Adipogenic induction significantly increased FITC intensity of OP-M cultures compared to their controls (*p* = 0.015). In addition, FITC intensity of the adipogenesis conditions of OP-M, S1 and of 2D cell culture were significantly higher than that of CA (*p* < 0.04). Microscopy revealed lipid droplet formation in adipose differentiation samples of OP-M, S1 and 2D culture. Cells grown as CA, without microcarriers, did not exhibit significant fat accumulation following adipose differentiation.

## Discussion

The rapid upscaling of CM production faces a significant bottleneck in the development of cost-effective, edible, and scalable scaffolding systems. Current commercial microcarriers, often derived from synthetic polymers or requiring complex coatings, present challenges regarding cost and the necessity for cell detachment prior to consumption. This study addresses these challenges by evaluating the ability of okara, a soy production by-product, to support cell proliferation and maintaining their differentiation potential. For this purpose, several types of okara based scaffolds were evaluated. We identified the types of soy microcarriers that supported AD-bMSCs’ viability better than others. Our findings suggest that agricultural waste has the potential to be successfully transformed into a high-value bio-scaffold that supports the proliferation of AD-bMSCs, and maintaining their differentiation potential, proposing a potential dual solution for waste reduction and sustainable meat production.

We tested eight types of soy microcarriers and found two types that supported cell viability best, scaffolds OP-FD and OP-M. Cell viability on these scaffolds was higher in comparison to cell viability as CA and comparable to commercial microcarriers. We decided to favor OP-M over OP-FD, which showed almost similar ability to support cell viability, even though OP-FD performed slightly better and, despite its longer shelf-life, because we think that lower price of manufacturing would be of a greater service to the CM industry. The iterative development of the soy-based microcarriers revealed that complex processing does not necessarily equate to better cell performance. While we initially hypothesized that treatments such as high homogenization, spray drying, or plasma treatment would enhance cell adhesion, our results show that the T4 variants (4RD, 4EA, 4HU) supported cell viability less than the OP variants (OP-FD, OP-DC-FD) that underwent a simpler freeze-drying method. These results made us change our initial strategy and test two types of okara with minimal treatment applied (OP-M and OP-T). Specifically, the OP-M microcarrier emerged as the optimal candidate. Not only did it support cell viability comparable to the commercial polystyrene-collagen standard (S1), but its manufacturing process was also significantly streamlined. Unlike other variants, OP-M did not require a final drying stage or pre-soaking before use, suggesting it could reduce industrial processing time and operational costs—critical factors for the economic feasibility of CM (Figure 1). However, OP-M might have a shorter self-life due to wet form delivery, hence the trades-off in cost vs. self-life and delivery must be considered at industrial setting. In addition, physicochemical characterization, including moisture content, batch-to-batch variability, and stability in culture medium, was not performed in this study and warrants future investigation.

After performing experiments in cell culture plates, we decided to examine the scaffolds’ ability to support cell viability in larger volumes. A set of preliminary experiments that better represent the industrial conditions (although still is much smaller than the actual volume that would be used in industry) was conducted, demonstrating the potential scalability of these soy microcarriers in larger culture volumes. In comparative long-term experiments using high volume flasks, the OP-M microcarriers consistently supported cell viability (Figures 3A–C; Supplementary Figures 4A,B). At the first time points of evaluation, there is a lag phase in cell growth which could be related to the big increment in volume. It is possible that higher seeding density is needed in higher volume growth and should be calibrated for future large-volume experiment. From day 17 onwards, OP-M scaffold in static condition outperformed other conditions, including the commercial S1 microcarriers. Notably, these promising results are preliminary, showing biological feasibility and a proof of concept, but need more calibrations and biological repeats to accurately assess the ability of OP-M to support cell viability in larger volumes.

Particle-size distribution is an important physical characteristic of particulate scaffolds because it influences scaffold packing, available surface area, and mass transport, all of which may affect cell–scaffold interactions. The present results demonstrate that particle size alone did not determine biological performance. The two scaffold formulations that best supported bMSC growth, OP-FD and OP-M, differed in both median particle size and particle-size distribution, indicating that no single particle-size profile was required for successful cell growth. However, the particle-size analysis presented here provides only preliminary physical characterization, as it was based on one sample from each scaffold batch and therefore does not provide comprehensive batch-to-batch validation. An additional practical advantage of the wet OP-M scaffold was its storage stability following sterilization. Although wet okara is inherently susceptible to microbial spoilage because of its high moisture and nutrient content, autoclave-sterilized OP-M remained sterile for at least 6 months of storage at 4 °C while retaining its ability to support cell growth. This finding demonstrates that wet okara-based scaffolds can be successfully stored after sterilization and highlights effective sterilization as a key requirement for their future production and application. Translating this wet-scaffold approach to an industrial scale will require continuous sterilization methods, such as UHT (50), as well as strict standardization of total solids and fiber profiles to address the inherent inter-batch compositional variability of wet agricultural by-products and facilitate the transition to a standardized food-grade ingredient. Because soy is a recognized major allergen and contains anti-nutritional factors such as phytates and trypsin inhibitors, processing parameters must be optimized to reduce the levels of these compounds (51). In addition, clear allergen labeling will be required for the final hybrid cultured meat product to ensure regulatory compliance and consumer safety.

An important point that should be noted is that OP-M scaffolds with stirring of the medium showed poor cell viability results in comparison to static conditions. This observation could imply that OP-M scaffolds are sensitive to shear forces that are found in bioreactors. Another possible explanation is that the lack of intermediate non-stirring periods, impair the ability of cells to expand from one microcarrier to another. This finding highlights the need for bioreactor protocols that are specifically tailored to the mechanical properties of plant-based scaffolds rather than simply adopting protocols designed for synthetic microcarriers. When cells are cultured in bioreactors, it is important to ensure sufficient movement of the medium, in order to provide enough oxygen and nutrients to all cells. If the shear forces indeed harm the OP-M scaffold, more gentle ways of mixing the medium should be considered. Further testing should be done with OP-M and OP-FD scaffolds in large volumes, with calibration of the appropriate medium stirring method, along with stirring strength and duration.

Crucially, the culturing on a soy-milk production-waste scaffold did not compromise the biological characteristics of the cells. We found that, AD-bMSCs expanded on novel materials retained their ability to proliferate and maintain fat accumulation in adipose differentiation protocol later in the process. Our characterization assays confirmed that cells recovered from OP-M microcarriers maintained typical fibroblast-like morphology, high proliferation rates, and the expression of characteristic mesenchymal surface markers CD44 and CD29 (Figure 4). This suggests that the okara-derived material provides a biocompatible surface that mimics the necessary physiological cues for AD-bMSCs maintenance. The cells expanded on soy microcarriers exhibited robust lipid accumulation upon induction, evidenced by BODIPY staining and flow cytometric analysis, with efficiency comparable to or marginally better than control conditions. It should be noted that, cell differentiation protocol in our experiments was performed on cells that re-adhered to plastic, in order to be able to better analyze the effect, as soy is autofluorescence in multiple wavelengths, including those that are used for quantification of fat content. Considering that one of the main advantages of the use of soy scaffolds is the elimination of the need to detach the cells from it, showing cell differentiation directly on scaffolds is essential. Future experiments should test cell adipogenesis directly on the scaffolds, and should consider studying also other cell types (such as bovine satellite cells- BSCs) to test myogenesis as well. Utilizing an edible scaffold that can remain part of the final product, eliminates the need for enzymatic dissociation, thereby preserving the extracellular matrix and adding to the textural quality of the final meat product.

Several studies described the potential of repurposing agricultural waste into scaffolds (30–36), most of the studies focus on cellulose rich materials that can serve as a good inter cellular structure support. Soy-pulp is both rich in cellulose and has additional nutritional values, particularly, its high protein content which enhance cell adhesion (30). Most studies, including those that produced and investigated soy derived scaffolds, reported complicated production protocols. Additionally, most of these studies show suitability of the scaffolds to human or mice cell types, which are less relevant to the CM industry. The potential to use soy-derived scaffold for CM was previously shown by Ben-Arye et al. as a successful agent for proliferation and differentiation of BSCs (42). Notably, cell culturing on microcarriers is more efficient than on a large scaffolding surface, for an essential need of the CM production- reaching a sufficient cell mass (6), and indeed BSCs were successfully grown on microcarriers (28, 29). In this study, we turned soy waste, using easy and cheap manufacturing procedures, into microcarriers that support cell culture growth. We show that another type of cells that hold beneficial properties to CM- AD-bMSC, is compatible with soy scaffolds. Thus, our results can lay the ground for culturing and differentiating bovine cells on soy microcarriers, and for co-culturing different cells (e.g., AD-bMSCs and BSCs) together for CM production.

Alamar Blue measures cellular metabolic activity rather than cell number directly, therefore, its signal may be influenced by differences in the physical and biochemical properties of the culture substrate. In the present study, cells were cultured on commercially available collagen-coated plastic microcarriers (S1), plant-derived okara microcarriers (OP-FD), and as CA, representing culture environments with distinct material compositions and mechanical properties. Since these parameters might affect metabolic activity, it was therefore important to verify that Alamar Blue truly reflected cell number across these different culture systems. Thereby, we conducted comparison experiment, evaluating same samples both with Alamar Blue and by DNA quantification using Qubit. Both methods showed similar results (Figures 2F,G). Cells grew significantly better on the scaffolds (OP-FD and S1) than as CA, demonstrating agreement between Alamar Blue and DNA-based Qubit results, establishing Alamar Blue as a valid method for cell quantification in scaffolds OP-FD and S1 under the tested conditions. The mean fold change of both scaffolds compared to CA, was higher in Alamar Blue than in Qubit measurements (in the 50,000 and 100,000 seeding densities, Supplementary Figure 3D). This can result from higher metabolic activity of cells on scaffolds, from higher rate of cell death in the CA condition, or from both. Furthermore, it seems that scaffold material has no influence on the fold change. These two methods complement each other, and provide a better description of cell state in every condition.

In this study, we measured 10-15-fold increase in total cell viability in 7 days culturing on scaffold OP-M, corresponding to a population doubling time of approximately 30–50 h (Figure 2). This is comparable to the approximate 50-h doubling time reported for bovine satellite cells cultured on dextran-based microcarriers (28, 29). Since bMSCs are generally expected to proliferate faster than satellite cells (52), there is likely room for further improvement; optimizing culture parameters may therefore reduce doubling time and support more efficient cell expansion.

In conclusion, this study demonstrates the potential of okara-derived soy microcarriers, particularly the OP-M variant, as a promising, sustainable alternative to synthetic scaffolds. By valorizing a food industry by-product, we have showed the feasibility of producing a scaffold that is edible, low-cost, and biologically effective for AD-bMSCs expansion without harming the cells’ biological characteristics. Future work will focus on optimizing production, culturing and bioreactor parameters to accommodate the specific physical characteristics of these organic carriers and investigating their potential to support co-cultures of myocytes and adipocytes, moving one step closer to a fully structured, slaughter-free meat product.

## Materials and methods

### Cells

AD-bMSCs were derived from a subcutaneous layer of 3-month-old female calf as described and characterized in Zirman et al. (49) For microscopy visualization of cell on scaffolds we used GFP-expressing bMSCs. Immortalized GFP-expressing bMSCs were a generous gift from Prof. Sharon Schlezinger (HUJI), prepared by introducing SV40T and hTERT, followed by lentivirus transduction of pLKO_047 vector (Broad Institute) and puromycin selection (53).

### AD-bMSCs culturing routine

AD-bMSCs were expanded in low-glucose DMEM supplemented with 13% fetal bovine serum (FBS, HI, Brazil origin, Gibco), 2 mM L-glutamine, and 100 units/mL Penicillin G sodium salt and 0.1 mg/mL Streptomycin sulfate, (Sartorius). Cells were maintained in polystyrene plates in a humidified incubator at 37 °C with 5% CO2 and passaged every 3–4 days at 60–80% confluency Trypsin–EDTA solution 0.25% (Gibco). Cell number was measured with automated cell counter (countess 4, Thermofisher).

### Microcarrier manufacturing

The soy-based raw material preparation followed a staged workflow to yield a functional, dry product suitable for scaffold fabrication. Initial raw material preparation involved washing dry whole soybeans to remove surface impurities, followed by grinding, hydration and another grinding step to facilitate the subsequent extraction process. The resulting mixture was collected, leading into the base processing stage, where the separation of the liquid fraction from the insoluble solids (soy pulp), known as okara, was achieved via decanting. Following separation and okara dilution, the various treatments stage commenced. The okara was diluted with DDW in a 1:10 (w/v) ratio before undergoing further property modification. The first step for most processed okara was drying (i.e., heat/spray/freeze), which efficiently removed residual moisture and yielded the dry, powder-form material. Next, the material was subjected to explicit treatments (e.g., grinding, homogenization, plasma, decellularization) to enhance its biological and mechanical characteristics as needed for the final scaffold application. The different scaffold types manufacturing was iterative, i.e., best performing scaffold were used for planning the next series of scaffold variations. Specifically, eight different scaffold types were manufactured in three series. The first series of microcarrier variations included double ground heat (oven) dried okara, termed here T4 scaffold (Figure 1B). Next, three additional treatments for the microcarrier were tested: high homogenization followed by spray drying (4RD), additional plasma treatment type 1 (Vaculab, 2 mbar, 150 s’, scaffold 4EA) and additional plasma treatment type 2 (Harrick Plasma Cleaner PDC-32G, scaffold 4HU). The second series of microcarrier variations included OP-FD- a freeze dried plain okara (Figure 1C) and OP-DC-FD- a decellularized plain okara followed by freeze-drying treatment. The third set of variations included OP-M (plain okara, in wet form, from soy-milk industry) and OP-T (plain okara, in its’ wet form, from tofu industry) that were only diluted with DDW in a 1:10 (w/v) ratio and autoclaved.

### Okara nutrient composition

The following tests were performed for assessing nutrient composition of okara. Sugar determination was performed using liquid chromatography with charged aerosol detection (HPLC-CAD). Samples were prepared by appropriate dilution in the mobile phase or compatible solvent, filtered through 0.45 μm filters, and injected directly into the HPLC system. Protein content was determined using the Dumas combustion method. This technique involves complete combustion of the sample at high temperature in an oxygen atmosphere, followed by reduction of combustion gasses and measurement of total nitrogen, using a thermal conductivity detector (TCD). The nitrogen content is then converted to protein content using an appropriate conversion factor. Fat content was determined using a rapid method combining microwave drying with Time Domain Nuclear Magnetic Resonance (TD-NMR). This technique measures the protons in liquid oil, providing direct quantification of fat content without the use of organic solvents. The method simultaneously determines total solids and fat content. The NMR signal from protons in the oil phase was recorded and fat content was calculated.

### Microcarrier addition to cells

Soy scaffolds (besides OP-M and OP-T) were received sterile and in a dry form. The scaffolds were soaked in DMEM 24 h before introducing to cells, at 0.005 g/mL (Figure 2A). OP-M and OP-T scaffolds were received wet (okara diluted 1:10 with DDW) and were autoclaved 20 min in 120 °C before introducing to cells at the same concentration of 0.005 g/mL, without pre-soaking. Sartorius SoloHill microcarriers (cat number: SK102-1521B-C221-020) were sterilized and prepared according to the manufacturer’s instructions and diluted to same concentration. 100 μL of scaffold solution was added to plates, then AD-bMSCs were added on top.

### Cell growth in 24-well plates

Wells were covered with FaCellitate (bioflat) to prevent adhesion of the cells to the wells. Scaffolds were added to the wells, then cells were added on top (seeding density-50,000 cells/well, passage 8–12). Cells were grown for 3–5 days, as described in ‘culturing routine’, and then, cell viability was measured using Alamar Blue (Resazurin sodium salt, Sigma-Aldrich) assay every 2–5 days. On measurement day, growth medium with scaffolds from each well were collected and transferred into new wells, to ensure that we measure viability of cells adherent to scaffolds. At the end of measurement, medium with AB was replaced with fresh medium, and cells were left for further growth.

### Cell growth in flasks

Glass Flasks were treated with Sigmacote (Sigma-Aldrich) to prevent cell adhesion to bottom. Scaffolds were added in final concentration of 0.005 g/mL, cells (passage 10 and 12) were seeded at 50,000 cell/mL. When indicated, the flask was placed on a magnetic stirrer, rotating at 80 rpm, or placed stationary in the incubator. In several time points a sample of 0.5 mL was taken from each flask, and Alamar Blue assay was performed, as described below.

### Cell viability measurements

Cell viability was measured using Alamar Blue assay. This assay enables long term experiments, as it does not kill the cells and allows to keep culturing the cells after the viability test, hence measuring the same specific cell culture over time (in contrast to seeding replicates for each evaluation point). First, each well content was transported into a new well, then, Alamar Blue reagent (Resazurin sodium salt, Sigma-Aldrich) was added into both old and new wells (in order to quantify viable cells that were transported with scaffolds to new wells and to check presence of leftover cells that were adherent to old wells). After 2 hrs of incubation, three sample replicates from each well were transported into 96-well plate and cell viability was evaluated by measuring fluorescence at 540 nm, using a plate reader (Synergy-HTX, along with the software GEN5 3.11, Bio-tec). For calculating cell viability from the measured fluorescence, blank measurements were subtracted from each sample. Blanks of each condition were incubated with the corresponding scaffold prior to measuring. In order to estimate the actual number of cells in each sample, a standard curve was calculated, using known amounts of cells that were incubated with the Alamar Blue reagent, cell number was determined using automated cell counter (countess 4, Thermofisher). In each cell viability experiment that was carried in cell culture plates we used three biological replicates for each condition and three technical repeats for each replicate. In viability tests that were performed on cell that grew in flasks, there was only one biological replicate and three technical repeats for each measuring point. To minimize the contribution of surface-adhered cells to the measurement, wells were treated with FaCellitate (bioflat) coating to prevent cell adhesion to the well surface. On measurement days, all well contents were transferred to new wells, and only scaffold-associated or suspended cells were quantified. The fraction of cells remaining adherent to old wells was measured separately and consistently found to below detection limit or redundant.

### Cell number estimation by DNA quantification

Cells were seeded on microcarriers OP-FD and S1 and as CA, in 24-well plates and grown for 3 days. Three seeding densities (25,000, 50,000, and 100,000 cells/well) were used, along with scaffolds concentration of 0.01 g/mL. On measurement day, growth medium with scaffolds from each well were collected and transferred into new wells and cell viability was measured in both transferred and original wells, using Alamar Blue. Next, content of both transferred and leftover wells was analyzed for total DNA content as proxy for cell quantity. For DNA quantification, samples were centrifuged, removing medium without pallet, while maintaining same volume in all samples. RLT buffer was added (at 1:2.5, sample: RLT) to each sample, placed in 50*C with gentle shaking for 20 min, then incubated overnight. DNA was measured using Qubit DNA Quantitation HS kit (Invitrogen). This experiment was performed in 3 biological repetitions, with 2 technical repetitions for Qubit (Qubit 4 Fluorometer, Invitrogen) and 3 technical repetitions for Alamar Blue. Cell quantity was calculated by dividing the total DNA measured by estimated DNA content (6.6 pg. per cell). Z scores for each seeding condition (separately for different seeding number on each scaffold type or as CA) were calculated both for Alamar Blue and Qubit results, and correlation between Z scores was calculated.

### Cell growth on 2D plates after proliferating in 3D

After a month of proliferating in flasks, on scaffolds (OP-M and S1) or as CA, samples of cells with scaffolds were obtained. Samples were treated with Trypsin–EDTA solution 0.25% (Gibco) with incubation time of 10 min to remove the cells from the scaffolds and filtered the cells through a 70 μm sieve filter, and seeded in 6-well plates (to promote adhesion). Estimated seeding density was 50,000 cells/mL. Cells were cultured for 4 days, then counted with cell counter. Three biological repeats were measured, averaging 4 technical repeats.

### Lipid accumulation

After showing the cells ability to grow well on soy scaffolds, we wanted to verify that the cells maintained their ability to differentiate into adipose cells. We took scaffolds (OP-M and S1) with cells that were adherent to them for a week, added Trypsin–EDTA solution 0.25% (Gibco) with incubation time of 10 min to remove the cells from the scaffolds, and filtered the cells through a 70 μm sieve filter. The cells were seeded in new cell cultured dishes, then, lipid accumulation and adipogenesis protocols were performed. For adipogenesis protocol, cells were seeded at 50,000 cells/well in 6-well plates, three biological replicates for each experimental condition. Cells that came off scaffolds and to naïve cells received adipogenesis induction medium for 14 or 18 (2 different batches) days with medium changes every 2–3 days. Induction medium contained 10% FBS, 1 μM dexamethasone, 0.5 mM IBMX, and 10 μg/mL insulin. Cells were stained using BODIPY and Hoechst and analyzed as described next, to compare lipid droplets content relative to control cells.

### Antibodies and staining

For MSCs surface markers assessment, cells were collected using Trypsin–EDTA solution 0.25% (Gibco) with short incubation time (of 5 min). Then, centrifuged at 1200 rpm for 5 min, and washed with DPBS (Sartorius) supplemented with 1% bovine serum albumin (BSA; Sigma Aldrich). The cell suspension was passed through a 70-μm nylon mesh filter. Cells were incubated with the following fluorophore-conjugated anti-mouse antibodies Anti-CD44 – FITC (Cat. no. GTX80087, GeneTex) and anti-CD29-APC (Cat. no. 303008, BioLegend), antibodies were diluted 1:1000 in DPBS (Sartorius), and incubated in dark for 30 min. Cells were washed twice with PBS, centrifuged at 500 rpm for 5 min, and finally resuspended in 1 mL PBS. Labeled cells were analyzed using a BD Accuri™ C6 Plus flow cytometer, recording fluorescence in the appropriate channels for each fluorochrome. Data acquisition and plot generation were performed using the BD Accuri C6 Plus software and analyzed using in house R script.

For adipogenic differentiation efficiency assessment we performed fluorescent staining. BODIPY 493/503 (Invitrogen) was prepared at 5 mM concentration and diluted 1:2000 in fresh growth media and incubated with cells for 30 min. Hoechst 33342 was prepared at 10 mg/mL concentration and diluted 1:2000 in fresh growth media and incubated for 10 min. Stained cells were analyzed using a BD Accuri™ C6 Plus flow cytometer, and BD Accuri C6 Plus software to quantify BODIPY-positive cells. For lipid droplets accumulation analysis, cells were stained with BODIPY and Hoechst in the same manner, and images were captured using fluorescence microscope (Nikon Eclipse Ti2) under identical exposure settings.

### Statistical analysis

The analysis was performed using excel (for t-test) and R for ANOVA. In most experiments, in order to compare viability of different conditions over time, a two-way ANOVA was followed by Tukey’s multiple comparison test. Viability of cells grown in plates, was tested using three biological repeats for each experimental condition, in addition, three technical repeats were obtained and averaged into each biological repeat. In the large volume experiments, there was one biological repeat per condition, which was measured in three technical repeats. In Alamar Blue vs. Qubit experiment, we performed a One-Way ANOVA followed by Tukey’s HSD post-hoc test (to adjust for multiple pairwise comparisons) on three biological repetitions, in order to determine if the differences between the growth surfaces are statistically significant at each seeding density. To analyze cell viability on plates after culturing in flasks we used Kruskal-Wallis nonparametric test, using three biological replicates and four technical repeats. Surface markers CD44 and CD29 measurements after growth in different conditions was analyzed with Welch’s one-way ANOVA followed by Games-Howell multiple comparisons test, there were six biological replicates in in all conditions, besides 2D culture with three biological replicates. A two-way ANOVA was run on the OA accumulation and adipogenesis experiments, followed by Tukey’s multiple comparison test. There were three biological repeats for every condition, except for controls of adipogenesis, that had only one repeat. T4 cytocompatibility assessment analysis was performed with 2-tailed student T-test on three biological replicates. Biorender and R were used for visualization.

## Data Availability

The original contributions presented in the study are included in the article/Supplementary material, further inquiries can be directed to the corresponding author/s.
