# Supplementary material for: Assessing the ability of bovine mesenchymal stem cells to grow on okara-based scaffolds derived from soy milk waste
Source: Front Nutr. 2026 Aug 31;13:1828885. doi: 10.3389/fnut.2026.1828885 (PMC13572135; doi:10.3389/fnut.2026.1828885)
Supplement: Supplementary file 1 [file Data_Sheet_1.docx]

Assessing the ability of bovine mesenchymal stem cells to grow on okara-based scaffolds derived from soy milk waste

**Orit Dash^1,2^, Maor Hay^3^, Mamoun Abed El-Nabi^1,4^, Shadi Tawil^2^, Sharon Schlesinger^2^, Kobi Meiri^3^, Roni Rak^1^**

^1^ Institute of Animal Sciences, Agricultural Research Organization - Volcani Institute, Rishon LeZion, Israel;

^2^ Department of Animal Sciences, Faculty of Agriculture, The Hebrew University of Jerusalem, Rehovot, Israel;

^3^ Tnuva – R&D department, Rehovot, Israel;

^4^ The Mina and Everard Goodman Faculty of Life Sciences, Bar-Ilan University, Ramat Gan, Israel;

# Supplementary Information

**Okara nutrient composition**

Okara composition was determined before the processing into the different scaffold types (supplementary table 1).

Sugar determination was performed using liquid chromatography with charged aerosol detection (HPLC-CAD). The isocratic elution profile with 85:15 ACN:H₂O at 60°C provided excellent separation of the target sugar compounds within the 22-minute analytical window. Peak identification was confirmed by comparison with retention times of authentic sugar standards. The amounts of all sugars in the samples were insignificant- resulting in less than 0.01 g in total in a 26 g sample.

Crude protein levels were determined using Dumas combustion method. Nitrogen peak profile resulting from sample combustion at 990°C was analyzed. The integrated area under the peak is directly proportional to the total nitrogen content in the sample. The sharp, symmetrical peak shape indicates complete combustion and efficient gas separation. The nitrogen content was then converted to protein content using a soy-based products conversion factor of 6.25 (a useful default factor according to Food and Agriculture Organization and to the World Health Organization), resulting in 34.6% of the total weight of a dry sample.

Fat determination using the CEM SMART Trac II system provided rapid, solvent-free quantification of lipid content in okara samples. The TD-NMR method measures the signal intensity from protons in the liquid oil phase, which correlates directly with fat content. Results were obtained within approximately 10 minutes per sample, including both the microwave drying phase for moisture determination and the NMR measurement phase for fat quantification. The method successfully provided both wet-basis and dry-basis fat content values, allowing for rapid compositional assessment. Fat content in okara samples was 19.2 % of the total weight of a dry sample.

Fiber content was not measured directly, but by calculating the difference between the total solids measured and the protein and fat measured. Measurements were obtained from the TD-NMR method which simultaneously determines total solids and fat content, and from Dumas combustion method which determined crude protein levels. Subtracting protein and fat levels from the total solids measured resulted in fiber content of 46.2 % of total weight.

| **Component** | **Weight [g]** | **% of dry weight** |
| --- | --- | --- |
| Insoluble Soy fibers | 12 | 46.2 |
| Insoluble soy proteins | 9 | 34.6 |
| Carbohydrates (sugars) | ~0 | 0 |
| Lipids | 5 | 19.2 |
| **Total** | **26** | **100** |

Supplementary table 1: Okara nutrient composition on a dry-weight basis.

**Scaffold characterization using particle size analysis**

Quantitative particle size distribution analysis was performed via laser diffraction using Mastersizer 3000+ (Malvern Panalytical). Laser diffraction analysis revealed distinct particle-size distributions among the okara-based scaffold formulations, demonstrating that scaffold processing, hydration, storage history, and manufacturing batch all influenced particle-size characteristics (Supplementary Figures 1A-D). Rehydration in DMEM increased the apparent particle size of the freeze-dried scaffolds, particularly OP-FD and OP-DC-FD, and generally broadened their particle-size distributions. The scaffold formulations exhibited a wide range of median particle sizes (Dv₅₀ = 109–649 µm); however, distribution width varied independently of Dv₅₀ and provided additional insight into material characteristics. OP-FD exhibited relatively narrow and homogeneous particle-size distributions across both production batches, whereas prolonged refrigerated storage markedly increased the particle-size heterogeneity of OP-M, resulting in a broad distribution with a pronounced coarse-particle tail despite a relatively small median particle size. These findings demonstrate that median particle size alone is insufficient to characterize scaffold morphology and highlight the importance of considering both particle size and distribution width when comparing particulate scaffold materials. Comparison of the volume-weighted mean diameter (D[4,3]) and the median particle diameter (Dv₅₀) revealed notable differences in the contribution of coarse particles among the scaffold formulations. Samples in which D[4,3] substantially exceeded Dv₅₀, particularly the long-stored OP-M scaffold and the rehydrated OP-FD and OP-DC-FD scaffolds, exhibited a greater contribution of large particles than indicated by the median particle size alone. In contrast, T4 and the fresh OP-M scaffolds displayed relatively small differences between D[4,3] and Dv₅₀, consistent with more homogeneous particle-size distributions. These observations were further supported by the PSD curves and the elevated Dv₉₀ and Dv₉₉ values measured for the same samples, emphasizing that median particle size alone does not fully describe scaffold particle-size characteristics.

**Supplementary Figure 1: Particle size characterization of okara-based scaffolds by laser diffraction.** Particle size distribution of freeze-dried and wet okara-based scaffolds was analyzed by laser diffraction. Freeze-dried scaffolds (T4, OP-FD, and OP-DC-FD) were analyzed before and after overnight rehydration in DMEM, whereas OP-M and OP-T were analyzed in their native wet state. Two independent production batches of OP-FD and OP-M were included, and an additional OP-M sample stored at 4°C for six months was analyzed to assess the effect of prolonged storage. **(A)** Volume-based particle size distribution curves. **(B)** Comparison of the volume-weighted mean diameter (D[4,3]) and the median particle diameter (Dv₅₀). **(C)** Characteristic particle diameters (Dv₁₀, Dv₅₀, and Dv₉₀). **(D)** Distribution width expressed as Span and Uniformity. Data is presented for n=1; D[4,3], volume-weighted mean diameter; Dv₁₀, Dv₅₀, Dv₉₀, Dv₉**_5_** and Dv₉₉, particle diameters below which 10%, 50%, 90%, and 99% of the cumulative particle volume is contained, respectively; Span = (Dv₉₀ − Dv₁₀)/Dv₅₀.

**Assessment of T4 microcarriers cytocompatibility**. AD-bMSCs were cultured in standard 2D conditions (cell control) or in the presence of T4 microcarriers physically separated from the cells using trans-well inserts (cells + T4 in inserts), preventing direct cell-microcarrier contact, but enabling soluble particles movement between compartments. Cell viability was quantified on day 5 after seeding, by Alamar Blue assay. The presence of the T4 microcarriers in inserts didn’t impair cell expansion, viability in both conditions didn’t differ significantly (Supplementary Figure 2A, p = 0.63).

**Measuring AD-bMSCs growth on commercial** **microcarriers.** To establish a positive control for comparison with the novel soy-based scaffolds, we measured the viability of AD-bMSCs on six microcarriers obtained from Sartorius (Sartorius SoloHill® Microcarriers starter kit, SK102-1521B-C221-020). Commercial microcarriers varied by material and surface modification: Collagen (S1, cross-linked polystyrene coated with gelatin), Hillex II (S2, modified cationic-charged polystyrene), Star-Plus (S3, cross-linked modified cationic-charged polystyrene), FACT III (S4, cross-linked cationic-charged polystyrene coated with gelatin), Plastic (S5, cross-linked polystyrene), and Plastic Plus (S6, cross-linked cationic-charged polystyrene). We compared the total cell viability of cells grown on microcarriers with plate adherent control cells (2D culture). Cells were cultured for eight days and their viability was measured twice (on days 3 and 8) with the Alamar Blue assay. Scaffold S1- ‘Collagen’- cross-linked polystyrene beads coated with gelatin, with size range of 125-212 µm showed best results. There was no significant difference in cell viability on the six scaffolds, on the two time points checked, but cell viability on scaffold S1 was the highest. Cell viability on scaffold S1, on day 8 was significantly higher in comparison to cell viability in 2D culture (Supplementary Figure 2B). This scaffold was chosen to serve as a control for comparing our cell viability on different types of Soy scaffolds.

**Concentration gradient experiment of OP-FD microcarriers**. We performed a scaffold concentration gradient experiment to determine the optimal concentration of OP-FD microcarriers for expanding AD-bMSCs. We used 3 different seeding densities (25K, 50K and 100K cells/well) and 3 different concentrations of OP-FD microcarriers (0.025, 0.05, 0.1 and 0.2 g/mL), were used in every seeding density, and compared to cells on S1 microcarrier and to cell growing in aggregates (CA). Cells were seeded in 24-well plates and 100 µL of each scaffold concentration were added to different wells. Viability was measured on days 3 and 6 after seeding, using Alamar Blue assay (Supplementary Figure 2C). To determine the optimal seeding density and OP-FD concentration for promoting cell proliferation, viability values were normalized to the initial seeding number to estimate relative expansion efficiency and compared to S1, which served as a reference scaffold. S1 supported the highest expansion efficiency across all densities (≈3.9× at 25k, 3.2× at 50k, 2.1× at 100k). None of the OP-FD concentrations exceeded S1 at this stage, although OP-FD 0.05 at 25k showed comparable expansion. At the second time point, significant main effects and interaction were again observed. Notably, OP-FD 0.05 demonstrated superior expansion efficiency at 50k and 100k seeding densities (≈5.6× and 3.25×, respectively), exceeding the corresponding S1 conditions (≈3.2× at 50k, 2.1× at 100k). OP-FD 0.025 also showed strong performance at moderate and high densities. These findings indicate a time-dependent enhancement of proliferative support by optimized OP-FD concentrations, particularly 0.05, at intermediate and high seeding densities.

Supplementary Figure 2: **System calibration: T4 cytocompatibility, commercial microcarrier screen and OP-FD concentration optimization.** A. Assessment of T4 microcarrier cytocompatibility. AD-bMSCs were cultured in standard 2D conditions (cell control) or in the presence of T4 microcarriers physically separated from the cells using trans-well inserts (cells + T4 in inserts). Cell viability on day 5 after seeding was quantified by Alamar Blue fluorescence; Sample specific blanks were subtracted from each sample, bars represent mean fluorescence and error bars indicate variability between replicates (n=3, students t-test, p=0.63). B. 6 Commercial microcarriers were screened for suitable positive controls for AD-bMSCs expansion. Cells were seeded in 24-well plates and their viability was quantified by Alamar Blue fluorescence on days 5 and 8. Relative cell viability is reported as % of the control cells- an adherent 2D cell culture, without microcarriers, bars represent mean fluorescence and error bars indicate variability between replicates. Sample specific blanks were subtracted from each sample. C. Optimization of OP-FD microcarrier concentration for AD-bMSC expansion. AD-bMSCs were cultured on the soy- based microcarrier OP-FD at varying scaffold concentrations (0.025, 0.05, 0.1, and 0.2 g/mL) and compared to cells grown as aggregates (CA) and to the commercial microcarrier S1. Estimated cell number was determined at two time points (day 3 and day 6), with bars representing mean values and error bars indicating variability between replicates. Sample specific blanks were subtracted from each sample.

**Evaluation of the Alamar blue as a valid method for cell viability comparison across conditions.** Alamar Blue was used throughout this study to assess cell viability on different scaffolds or as cell aggregates (CA). Here, we evaluate whether its metabolic readout accurately reflects cell number across the tested culture conditions. To this end, AD-bMSCs were seeded in 24-well plates, on OP-FD and S1 microcarriers, or cultured as CA at three seeding densities (25,000, 50,000, and 100,000 cells/well). After 3 days of culture, growth medium with scaffolds from each well were collected and transferred into new wells and cell viability was measured in both new (transferred) and old (leftover) wells using Alamar Blue. Total cell number was independently determined by DNA quantification using the Qubit assay (Qubit 4 Fluorometer, Invitrogen) on the corresponding samples.

To enable direct comparison between the two methods, Alamar Blue and Qubit measurements were normalized as Z-scores within each scaffold type and seeding density. A strong linear relationship was observed between the two assays for all culture conditions (Supplementary Figures 3A–C). Correlation coefficients were ≥0.99, with regression slopes ranging from 1.02 to 1.11, indicating excellent agreement between metabolic activity and DNA-based cell quantification regardless of scaffold type.

Two-way ANOVA, followed by Tukey's HSD post-hoc test for multiple comparisons, was performed to compare Alamar Blue to Qubit results across conditions, there was no significant difference between the two methods (Supplementary Figure 3D, p>0.05). There was an insignificant but obvious trend showing that the fold change in Alamar Blue measurements in seeding densities 50,000 and 100,000 cells/well was higher than in Qubit measurements.

These findings support the use of the Alamar Blue assay as a reliable method for cell growth assessment on the scaffold types evaluated in this study.

Supplementary Figure 3: Comparison of Alamar blue and Qbit results of bMSCS growth on okara-based microcarriers. A–C. Correlation between cell viability measured by Alamar Blue and DNA-based cell quantification using Qubit assay, for cells cultured for 3 days on S1 (A), OP-FD (B), and as CA (C). Cells were seeded at 25,000, 50,000, or 100,000 cells/well, with scaffolds density of 0.01 g per well. Alamar Blue and Qubit measurements were normalized to Z-scores within each culture condition. Dashed lines indicate linear regression, and shaded areas represent the 95% confidence interval. Regression slope and Pearson's correlation coefficient (R) are shown for each condition. Data points represent mean ± SE of four biological replicates (n=4). D. Evaluation of Alamar Blue (AB) method by comparing it to DNA quantification using Qubit (same data as in Figures 2F-G). Cell viability on OP-FD, S1 and as CA was measured, after 3 days of culture. Sample specific blanks were subtracted from each sample, AB measures are expressed as fold change relative to the corresponding CA condition for each seeding density (25,000, 50,000, and 100,000 cells/well). Bars represent mean ± SD, symbols indicate individual biological replicates, each with three technical measurements (n=3, two-way ANOVA, p>0.05).

**Culturing large volumes of AD-bMSCs using soy-milk waste based microcarriers: Vessel variation.** We ran another round of the culturing large volumes experiment, with same seeding and growth medium volumes, but in a smaller volume glass flask (Supplementary Figures 4A-B). We seeded cells (50,000 cells/mL) on OP-M and S1 microcarriers, in 20 mL of growth medium, in 100 mL glass flasks. The cell viability was evaluated 5 times during 18 days of growth (on days 3, 6, 10, 14 and 18) using alamar blue. Again, cells grew significantly better on OP-M microcarriers, in the flask without the spinning of the medium. When comparing cell growth on OP-M without medium spinning and on S1, at the 2 first timepoints the cell viability on S1 was better (*p* < 0.0001), but from day 10 cell viability was significantly higher on OP-M than on S1 (*p* < 0.01).

Supplementary Figure 4: Cell viability over time in glass flasks was evaluated. A. Cell expansion over time in in 100 mL lab glass bottles on OP-M (our chosen soy microcarrier) in comparison to S1 and to CA.) was evaluated. Cells were grown for 18 days, and viability was evaluated five times, using Alamar Blue assay. Sample specific blanks were subtracted from each sample. values of fluorescence were normalized to initial cell seeding of 50,000 cells/mL (n=1, with three technical repeats). B. Photo of the 100 mL lab glass bottles that were used for culturing large volumes of ad-bMSCs. C. Left: cells came off scaffold OP-M, 2 weeks after reseeding in 100 cm^2^ dish. Right: cells migrated from CA and re-attached to cell culture plates, 2 weeks after reseeding in 100 cm^2^ dish. (x4, bar=100 µm).

**Oleic acid accumulation after proliferation on scaffolds**. For lipid accumulation ability assessment, we used OA. We took cells that grew on the soy microcarriers, on commercial scaffolds and cells that grew as CA and re-adhered to cell culture plates (Supplementary Figure 4C). Cells were seeded at 8300 cells/well in 12-well plates, 3 biological replicates for each experimental condition. OA at 500 mM concentration and was diluted 1:1000 with fresh medium and added to cells that came off scaffolds and to naïve cells. One day after administration of OA, cells were stained using BODIPY and Hoechst, then, lipid droplets content relative to control cells was measured by flow cytometric analysis and compared (Supplementary Figures 5A-B). Even though, the experiment didn’t show significant results, it showed that the cells maintained the ability to accumulate fat, and led us to proceed to the adipogenesis experiment.

Supplementary Figure 5: **Oleic acid accumulation after proliferation on scaffolds.** A. Box plots show BODIPY fluorescence intensity normalized by size (FCS) of cells that received OA in the growth medium, and their controls, one day after OA administration (n=3, two-way ANOVA, p>0.05). B. Histograms of FITC intensity normalized by cell size, for each condition. OP-MA, S1 and CA indicate cells that previously grew on scaffold or as CA, ‘Scaffold’ represents OP-M scaffold without cells that was stained with BODIPY, to assess the effect of the stain on the scaffold itself.
